# Ostial lesion of the anterior descending coronary artery treated via Szabo technique supported by stent boost imaging: a case report

**DOI:** 10.1186/s13019-021-01516-9

**Published:** 2021-05-17

**Authors:** Guangliang Wang, Xuemei Wu

**Affiliations:** 1 Department of Cardiology, Jiren Hospital of Far Eastern Horizon, Anda, P. R. China; 2 Department of Pediatric Neurology, First Hospital of Jilin University, 1 Xinmin Street, Changchun, 130000 Jilin Province China

**Keywords:** Szabo, StentBoost, Ostial lesion of the anterior descending coronary artery

## Abstract

**Background:**

Stenosis at the opening and bifurcation of the anterior descending branch and circumflex branch around the end of the left main trunk is difficult to repair. Accurate positioning of a stent is the key problem.

**Case presentations:**

Here we report the case of a 61-year-old man who suffered from paroxysmal chest pain for 1 year, without history of diabetes or hypertension. The coronary computed tomography showed mixed plaques in the proximal part of the anterior descending artery, with stenosis severe at 80–90%. The emergency coronary angiography showed occlusion of the anterior descending artery. During percutaneous coronary intervention, a drug-eluting stent was implanted into the anterior descending artery using the Szabo technique, supported by stent boost (StentBoost) imaging to pinpoint the location of the lesion. The patient’s paroxysmal chest pain was relieved after the procedure.

**Conclusion:**

We used StentBoost to verify the accuracy of stent placement and the Szabo technique to rectify long-term coronary stenosis, which achieved satisfactory results. Combining the Szabo technique with StentBoost imaging was helpful to accurately evaluate the area and locate the stent when treating this ostial lesion of the anterior descending coronary artery.

## Background

An ostial lesion of the coronary artery lies within 3 mm of the opening of the coronary artery [1]. The lesion is not easy to expand, as it is rich in elastic fiber tissue and easily retracts, and the rate of restenosis and calcification is high. After expansion, dissection can easily occur [2], and the consequences of complications are serious. Therefore, accurately positioning the stent to ensure a stable open artery has become an important concern of interventional physicians [3]. The bifurcation of the anterior descending branch and the circumflex branch lesions at the end of the left main trunk are always difficult to repair.

In 2005 Szabo et al. [4] proposed the use of two guidewires, one for insertion through the stent capsule, and the other through the end mesh of the stent for anchorage. This is referred to as the Szabo technique [4, 5]. Although wide application of the Szabo technique has significantly reduced the rate of restenosis, it can still occur [6].

Incomplete stent expansion and poor adherence are important risk factors of restenosis or thrombosis. To reduce these possibilities, stent boost (StentBoost) is an angiography imaging technology based on X-ray fluoroscopy that aids visualization of the stent, and thus to identify incomplete stent expansion and poor adherence [7, 8]. StentBoost shortens operative time and is easy to learn; operators do not require specialized training, and there are no additional costs to the patients.

Herein, we report the case of a limited lesion of the left anterior descending (LAD) artery treated with the Szabo technique aided by StentBoost imaging, in which the patient achieved a relatively satisfactory prognosis. We hope this article will help make interventional doctors aware of technology to accurately locate and repair a lesion of the LAD with a single stent.

## Case presentation

A 61-year-old man was admitted on 12 June 2017 with paroxysmal chest pain of 1-year duration, which had aggravated for the previous 2 months. Preceding these problems he was healthy with no history of diabetes or hypertension. Physical examination revealed blood pressure of 142/57 mmHg. The heart rate was 67 bpm, and the rhythm was regular. There was no obvious murmur or pericardial friction sound in any valve auscultation area. No abnormality was found in the routine examination of blood, urea and stool, liver function, kidney function, blood lipid, blood glucose, ion, blood coagulation, and thromboelastogram. The electrocardiogram showed that ST segment depression was 0.1 mv in V4, and the T wave was flat in V4–6. On coronary computed tomography, mixed plaques were observed in the proximal part of the LAD, with severe stenosis of 80 to 90%.

The experimental protocol was established in accordance with the ethical guidelines of the Helsinki Declaration and was approved by the Ethics Committee of Jiren Hospital of Far Eastern Horizon, China. Written informed consent was obtained from the patient.

**Fig. 1:**
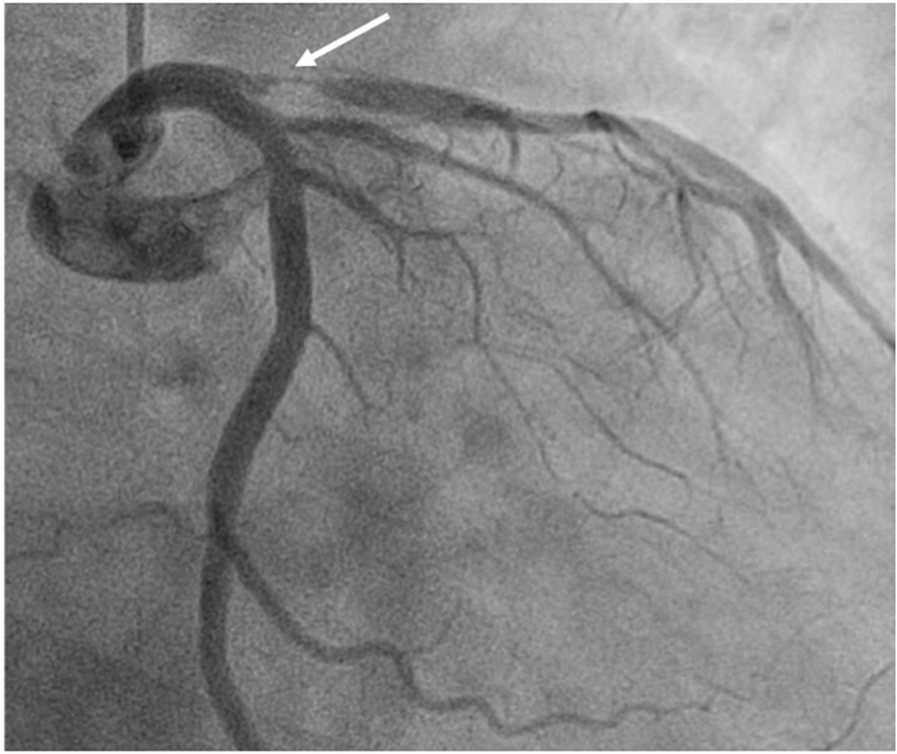
Coronary angiography displays severe stenosis of the opening of the anterior descending artery

**Fig. 2:**
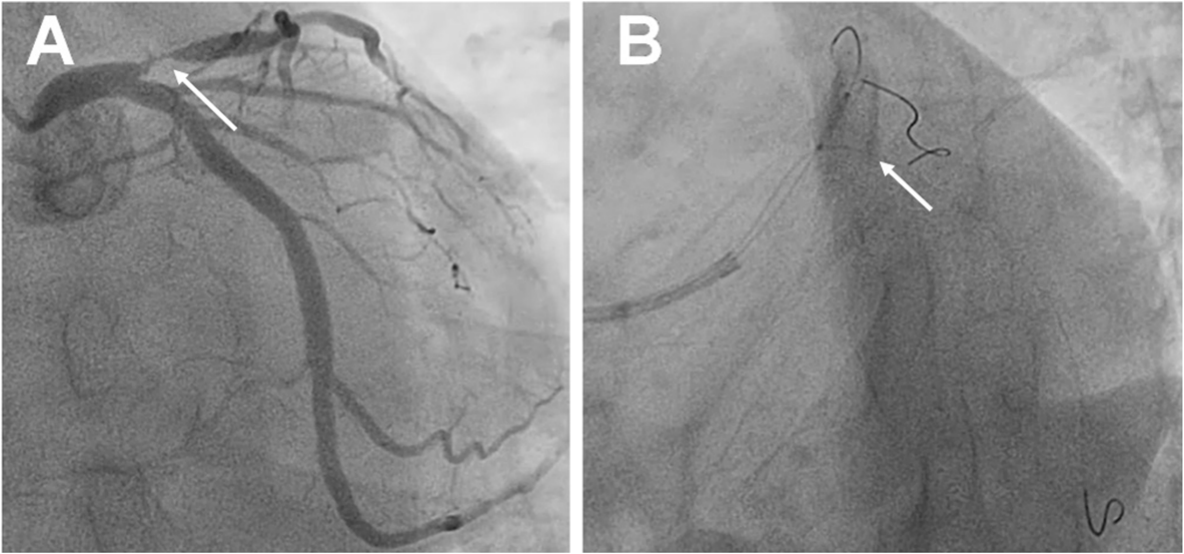
The stent was located using the Szabo technique. **a** After the stent was placed, the guidewire of the circumflex branch penetrated through the last mesh with slight expansion at the proximal end of the stent. **b** The opening of the anterior descending branch was accurately located. It can be seen that the angle of the guidewire of the circumflex branch was nearly 90 degrees at the last mesh of the proximal end of the stent

**Fig. 3:**
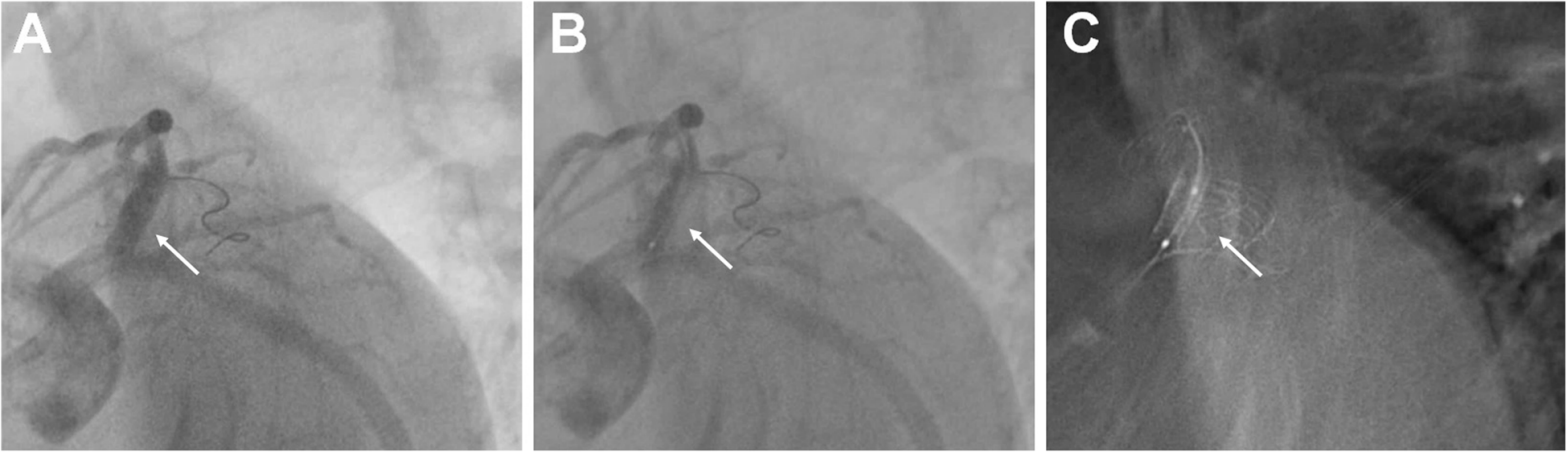
After the stent was released, StentBoost imaging was used to confirm the position of the stent at the opening of the anterior descending branch. Subfigures illustrate the steps of the StentBoost protocol. **a** The balloon was used as reference mark after the stent was released. **b** The proximal mark of the posterior balloon was located at the opening of the anterior descending branch. Angiography showed that the guidewire of the circumflex branch was close to the side of the anterior descending branch of the circumflex branch. **c** The proximal end of the stent covers the opening of the anterior descending branch and ends at the edge of the circumflex branch with reference to the guidewire of the circumflex branch

Aspirin (300 mg; Bayer, Germany) and clopidogrel (300 mg; Sanofi, France) were given. Enoxaparin (0.4 mL, bid; Sanofi, France) was injected subcutaneously. The emergency coronary angiography showed occlusion of the LAD. During percutaneous coronary intervention (PCI), a drug-eluting stent (3.5 mm in diameter and 12 mm in length) was implanted into the LAD (Figs. 1, 2 and 3). After the PCI, aspirin (100 mg qd) and clopidogrel (75 mg qd) were taken regularly.

## Discussion and conclusions

In the present case, during the procedure the stent was accurately positioned into the opening of the proximal part of the anterior descending branch using the Szabo technique. Vuurmans et al. [9] reported a case in which StentBoost imaging revealed previous lesions in the right coronary artery orifice; the proximal stent did not completely cover the orifice, leading to in-stent restenosis. This suggests that the StentBoost technique can be used to predict the long-term prognosis of coronary artery disease after stenting. Jin et al. [10] reported that StentBoost technology enabled evaluation of stent adherence and positioning, and stent release positioning. The X-ray radiation dose with StentBoost was comparable to that of normal conventional angiography positioning.

Since the clinical application of percutaneous transluminal coronary angioplasty (PTCA) in 1977, the therapeutics of coronary heart disease has undergone a revolutionary change, and many patients have been treated effectively [11]. However, rectification of ostial lesions has not been as satisfactory compared with other lesions, and the early success rate is only 60 to 84% [12]. The rate of important complications such as death and myocardial infarction was reported at 5%, which was higher than that of other lesions, and the restenosis rate was 46 to 61%; thus the effect of PTCA alone was poor [13]. With the introduction of the metal stent, acute complications such as dissection or vascular occlusion have been significantly reduced. While the immediate effect appears ideal, the long-term restenosis rate is 26 to 40% [14]. In particular, in the treatment of ostial lesions, if the stent protrudes into the left main artery, the risk of reoccurrence is high [15]. Thus, accurately locating and releasing the stent has become the focus of difficulties in treating such lesions.

The reader should note that, while the Szabo technique effectively treats ostium lesion of the coronary artery, it requires more surgical equipment and greater surgical skills compared with the traditional simple positioning with a single guidewire. In addition, this method increases the possibility of stent roughness and unloading. Therefore, the Szabo technique is reserved for selected appropriate cases [5].

The present study involves only a single case, with a short follow-up time, and many potential problems are unclear. For example, it is necessary to pre-adjust the stent using the hands, to fix and pinch it. This increases the risk of contamination and coating damage, and there is a question whether this may affect the release of coated drugs after implantation [16]. In addition, the function of the anchoring guidewire is to pull the stent and prevent it from inserting too far into the main branch, but in the process of pushing the stent, the guidewire will certainly compromise the stent coating. It is undecided whether this could increase the long-term risk of restenosis or thrombosis [5, 16]. Furthermore, there is concern regarding the last ring steel beam of the low-pressure expandable stent, and whether lifting it to make the anchor wire pass through the mesh of the stent, however gently, will inevitably change the geometry of the stent, leading to later clinical events [11]. Finally, although the drug-eluting stent represents great progress compared with percutaneous transluminal coronary angioplasty and bare metal stent implantation, restenosis is still possible inside the stent [17, 18]. These problems warrant further investigation.

The current risk factors of restenosis inside the stent [19, 20] include clinical characteristics, such as diabetes; pathological features related to small vessel, diffuse, and chronic occlusive diseases; and operative factors including insufficient stent expansion, poor adherence, and stent fracture. Preventing stent restenosis requires good stent adherence and complete stent expansion. The gold standard for evaluating stent implantation is intravascular ultrasound (IVUS), which accurately quantifies stent expansion [21, 22]. Unfortunately, at this time many hospitals in China lack an IVUS system, as the equipment is expensive and requires additional training of special operators [23].

StentBoost technology significantly enhances the visibility of stents during surgery, and assists accurate positioning, especially for the newer drug-eluting stents that are relatively thin and difficult to see [7, 24]. StentBoost can also show incomplete stent dilatation and vascular calcification, and guide the interventional treatment of bifurcation lesions [25]. It is reported that StentBoost and IVUS are interchangeable for achieving the stent expansion [8, 26].

A lesion of the coronary artery ostium is not easy to expand. After application of the Szabo technique, the precise position of the stent, choice of further treatment, and the probability of long-term restenosis may rely on StentBoost imaging. Through the image, the position of the stent can be verified, and the opening of the circumflex branch is not affected. Although IVUS can also evaluate the accuracy of the stent placement, the cost is higher. The StentBoost technology is easy to learn and will gain acceptance by interventionists, without increasing cost.

## Data Availability

The datasets used and/or analysed during the current study are available from the corresponding author on reasonable request.

## Abbreviations

LAD: Left anterior descending
PTCA: Percutaneous transluminal coronary angioplasty
IVUS: Intravascular ultrasound
